# Identification of physicochemical selective pressure on protein encoding nucleotide sequences

**DOI:** 10.1186/1471-2105-7-148

**Published:** 2006-03-16

**Authors:** Wendy SW Wong, Raazesh Sainudiin, Rasmus Nielsen

**Affiliations:** 1 Department of Biological Statistics and Computational Biology, Cornell University, Ithaca, NY 14853, USA; 2 Department of Mathematics, Cornell University, Ithaca, NY 14853, USA; 3 Wellcome Trust Sanger Institute, Wellcome Trust Genome Campus, Hinxton, Cambridge CB10 1SA, UK; 4 Department of Statistics, University of Oxford, 1 South Parks Road, Oxford OX1 3TG, UK; 5 Center for Bioinformatics and Department of Biology, University of Copenhagen, Universitetsparken 15, 2100 Kbh Ø, Denmark

## Abstract

**Background:**

Statistical methods for identifying positively selected sites in protein coding regions are one of the most commonly used tools in evolutionary bioinformatics. However, they have been limited by not taking the physiochemical properties of amino acids into account.

**Results:**

We develop a new codon-based likelihood model for detecting site-specific selection pressures acting on specific physicochemical properties. Nonsynonymous substitutions are divided into substitutions that differ with respect to the physicochemical properties of interest, and those that do not. The substitution rates of these two types of changes, relative to the synonymous substitution rate, are then described by two parameters, *γ *and *ω *respectively. The new model allows us to perform likelihood ratio tests for positive selection acting on specific physicochemical properties of interest.

The new method is first used to analyze simulated data and is shown to have good power and accuracy in detecting physicochemical selective pressure. We then re-analyze data from the class-I alleles of the human Major Histocompatibility Complex (MHC) and from the abalone sperm lysine.

**Conclusion:**

Our new method allows a more flexible framework to identify selection pressure on particular physicochemical properties.

## Background

Traditionally, the nonsynonymous to synonymous rate ratio (the *d*_N_/*d*_S _ratio, also known as *ω*) is a measure of the strength of selection acting on a protein coding nucleotide sequence. When the nonsynonymous substitution rate is higher than the synonymous substitution rate at a particular codon site, this site is assumed to be undergoing positive selection, i.e. selection in favor of new nonsynonymous mutations. Conversely, if the nonsynonymous substitution rate is lower than the synonymous substitution rate at a codon site, it is interpreted as evidence for negative selection, i.e. selection against mutations.

It is well-known that the rate of substitution between amino acids at a particular site depends on both its location in the protein (e.g. [1]) and the physicochemical properties of the amino acids involved (e.g. [2-6]). Site specific models have been successful in identifying particular residues, or codon sites, that have been targeted by positive selection (e.g. [7-9]). However, relatively little has been known regarding the particular physicochemical properties that have been subject to positive selection. Site-specific information regarding the physicochemical properties that have been subject to positive selection is of great interest in many systems. For example, in viral genes, site-specific information regarding physicochemical properties targeted by selection may shed evolutionary light on the biochemistry underlying mutational evasion of an immune response (e.g. [10]). Therefore, it would be very helpful to have statistical methods that can determine the physicochemical properties subject to selection at specific sites.

Sainudiin et al. (2005) [11] conducted a study to detect selection acting on the physicochemical properties at individual codon sites using the maximum likelihood framework by [12] and [13,11] generalized the concept of the nonsynonymous to synonymous rate ratio by replacing it with the property-altering to property-preserving rate ratio (*γ*) that is invoked by a partition of the amino acids according to the physicochemical properties of interest, to explore the physicochemical properties that were targeted by positive selection. A major limitation of their model is that it divided substitutions into two groups: (1) synonymous mutations and property conserving nonsynonymous mutations, and (2) property altering nonsynonymous mutations.

In this paper, we generalize the idea proposed by [11] by allowing three categories of mutations: synonymous mutations, property conserving nonsynonymous mutations, and property altering nonsynonymous mutations. The rate of the latter two types of mutations, each of which is scaled relative to the rate of synonymous mutations, is denoted by *ω *and *γ*, respectively. This allows us to investigate the selective pressure acting on any physicochemical property of interest using *γ*, while simultaneously accounting for the non-specific selective pressure at the amino acids level using *ω*. We also develop a set of new mixture models that allows site-specific inferences of selection acting on specific physicochemical properties. Finally, we illustrate the method on both simulated and real data sets.

## Results

### Implementation

#### Modeling physicochemical pressure

In order to test whether there is selective constraint or preference acting on a particular physicochemical property at the amino acid level, we introduce a new parameter *γ *within the framework of the continuous-time Markov chain models of codon evolution proposed by [14] and [15]. The state space of the model, assuming the universal genetic code, is given by the 61 sense codons. The 61 × 61 rate matrix *Q *= {*q*_*ij*_} gives the rate of transition from codon *i *to codon *j*. The transition rate from codon *i *to codon *j *is assumed to be proportional to the stationary distribution of codon *j*. Here we propose two modifications to the basic model to allow selection to act on specific physicochemical properties.

In Model A, if codon *i *and *j *differ by exactly one nucleotide, then

qij={πj          if i≠j by a synonymous transversionκπj       if i≠j by a synonymous transitionγijωijπj        if i≠j by a nonsynonymous transversion  γijωijκπj     if i≠j by a nonsynonymous transition            where {γij = 1 and ωij = ω, if i and j have the same physiochemical propertyγij = γ and ωij = 1, if i and j have different physiochemical properties     (1)
 MathType@MTEF@5@5@+=feaafiart1ev1aaatCvAUfKttLearuWrP9MDH5MBPbIqV92AaeXatLxBI9gBaebbnrfifHhDYfgasaacH8akY=wiFfYdH8Gipec8Eeeu0xXdbba9frFj0=OqFfea0dXdd9vqai=hGuQ8kuc9pgc9s8qqaq=dirpe0xb9q8qiLsFr0=vr0=vr0dc8meaabaqaciaacaGaaeqabaqabeGadaaakeaajugybuaabeqabiaaaqaabeGcbaqcLbwacqWGXbqCkmaaBaaaleaajugybiabdMgaPjabdQgaQbWcbeaajugybiabg2da9OWaaiqaaeaajugybuaabaqaeeaaaaGcbaacciqcLbwacqWFapaCkmaaBaaaleaajugybiabdQgaQbWcbeaajugybiabbccaGiabbccaGiabbccaGiabbccaGiabbccaGiabbccaGiabbccaGiabbccaGiabbccaGiabbccaGiabbMgaPjabbAgaMjabbccaGiabdMgaPjabgcMi5kabdQgaQjabbccaGiabbkgaIjabbMha5jabbccaGiabbggaHjabbccaGiabbohaZjabbMha5jabb6gaUjabb+gaVjabb6gaUjabbMha5jabb2gaTjabb+gaVjabbwha1jabbohaZjabbccaGiabbsha0jabbkhaYjabbggaHjabb6gaUjabbohaZjabbAha2jabbwgaLjabbkhaYjabbohaZjabbMgaPjabb+gaVjabb6gaUbGcbaqcLbwacqWF6oWAcqWFapaCkmaaBaaaleaajugybiabdQgaQbWcbeaajugybiabbccaGiabbccaGiabbccaGiabbccaGiabbccaGiabbccaGiabbccaGiabbMgaPjabbAgaMjabbccaGiabdMgaPjabgcMi5kabdQgaQjabbccaGiabbkgaIjabbMha5jabbccaGiabbggaHjabbccaGiabbohaZjabbMha5jabb6gaUjabb+gaVjabb6gaUjabbMha5jabb2gaTjabb+gaVjabbwha1jabbohaZjabbccaGiabbsha0jabbkhaYjabbggaHjabb6gaUjabbohaZjabbMgaPjabbsha0jabbMgaPjabb+gaVjabb6gaUbGcbaqcLbwacqWFZoWzkmaaBaaajuaGbaqcLbwacqWGPbqAcqWGQbGAaKqbagqaaKqzGfGae8xYdCNcdaWgaaqcfayaaKqzGfGaemyAaKMaemOAaOgajuaGbeaajugybiab=b8aWPWaaSbaaSqaaKqzGfGaemOAaOgaleqaaKqzGfGaeeiiaaIaeeiiaaIaeeiiaaIaeeiiaaIaeeiiaaIaeeiiaaIaeeiiaaIaeeiiaaIaeeyAaKMaeeOzayMaeeiiaaIaemyAaKMaeyiyIKRaemOAaOMaeeiiaaIaeeOyaiMaeeyEaKNaeeiiaaIaeeyyaeMaeeiiaaIaeeOBa4Maee4Ba8MaeeOBa4Maee4CamNaeeyEaKNaeeOBa4Maee4Ba8MaeeOBa4MaeeyEaKNaeeyBa0Maee4Ba8MaeeyDauNaee4CamNaeeiiaaIaeeiDaqNaeeOCaiNaeeyyaeMaeeOBa4Maee4CamNaeeODayNaeeyzauMaeeOCaiNaee4CamNaeeyAaKMaee4Ba8MaeeOBa4MaeeiiaaIaeeiiaacakeaajugybiab=n7aNPWaaSbaaKqbagaajugybiabdMgaPjabdQgaQbqcfayabaqcLbwacqWFjpWDkmaaBaaajuaGbaqcLbwacqWGPbqAcqWGQbGAaKqbagqaaKqzGfGae8NUdSMae8hWdaNcdaWgaaWcbaqcLbwacqWGQbGAaSqabaqcLbwacqqGGaaicqqGGaaicqqGGaaicqqGGaaicqqGGaaicqqGPbqAcqqGMbGzcqqGGaaicqWGPbqAcqGHGjsUcqWGQbGAcqqGGaaicqqGIbGycqqG5bqEcqqGGaaicqqGHbqycqqGGaaicqqGUbGBcqqGVbWBcqqGUbGBcqqGZbWCcqqG5bqEcqqGUbGBcqqGVbWBcqqGUbGBcqqG5bqEcqqGTbqBcqqGVbWBcqqG1bqDcqqGZbWCcqqGGaaicqqG0baDcqqGYbGCcqqGHbqycqqGUbGBcqqGZbWCcqqGPbqAcqqG0baDcqqGPbqAcqqGVbWBcqqGUbGBcqqGGaaicqqGGaaiaaaakiaawUhaaaqaaKqzGfGaeeiiaaIaeeiiaaIaeeiiaaIaeeiiaaIaeeiiaaIaeeiiaaIaeeiiaaIaeeiiaaIaeeiiaaIaeeiiaaIaee4DaCNaeeiAaGMaeeyzauMaeeOCaiNaeeyzauMaeeiiaaIcdaGabaqcLbwaeaqabOqaaKqzGfGae83SdCMcdaWgaaqcbawaaKqzGfGaemyAaKMaemOAaOgajeaybeaajugybiabbccaGiabb2da9iabbccaGiabbgdaXiabbccaGiabbggaHjabb6gaUjabbsgaKjabbccaGiab=L8a3PWaaSbaaKqaGfaajugybiabdMgaPjabdQgaQbqcbawabaGccqqGGaaijugybiabb2da9iabbccaGiab=L8a3jabbYcaSiabbccaGiabbMgaPjabbAgaMjabbccaGiabdMgaPjabbccaGiabbggaHjabb6gaUjabbsgaKjabbccaGiabdQgaQjabbccaGiabbIgaOjabbggaHjabbAha2jabbwgaLjabbccaGiabbsha0jabbIgaOjabbwgaLjabbccaGiabbohaZjabbggaHjabb2gaTjabbwgaLjabbccaGiabbchaWjabbIgaOjabbMha5jabbohaZjabbMgaPjabb+gaVjabbogaJjabbIgaOjabbwgaLjabb2gaTjabbMgaPjabbogaJjabbggaHjabbYgaSjabbccaGiabbchaWjabbkhaYjabb+gaVjabbchaWjabbwgaLjabbkhaYjabbsha0jabbMha5bGcbaqcLbwacqWFZoWzkmaaBaaajeaybaqcLbwacqWGPbqAcqWGQbGAaKqaGfqaaKqzGfGaeeiiaaIaeeypa0JaeeiiaaIae83SdCMaeeiiaaIaeeyyaeMaeeOBa4MaeeizaqMaeeiiaaIae8xYdCNcdaWgaaqcbawaaKqzGfGaemyAaKMaemOAaOgajeaybeaakiabbccaGKqzGfGaeeypa0JaeeiiaaIaeeymaeJaeeilaWIaeeiiaaIaeeyAaKMaeeOzayMaeeiiaaIaemyAaKMaeeiiaaIaeeyyaeMaeeOBa4MaeeizaqMaeeiiaaIaemOAaOMaeeiiaaIaeeiAaGMaeeyyaeMaeeODayNaeeyzauMaeeiiaaIaeeizaqMaeeyAaKMaeeOzayMaeeOzayMaeeyzauMaeeOCaiNaeeyzauMaeeOBa4MaeeiDaqNaeeiiaaIaeeiCaaNaeeiAaGMaeeyEaKNaee4CamNaeeyAaKMaee4Ba8Maee4yamMaeeiAaGMaeeyzauMaeeyBa0MaeeyAaKMaee4yamMaeeyyaeMaeeiBaWMaeeiiaaIaeeiCaaNaeeOCaiNaee4Ba8MaeeiCaaNaeeyzauMaeeOCaiNaeeiDaqNaeeyAaKMaeeyzauMaee4CamhaaOGaay5EaaaaaeaajugybiaaxMaacaWLjaGcdaqadaqaaKqzGfGaeGymaedakiaawIcacaGLPaaaaaaaaa@15D4@

Similarly in Model B, if codon *i *and *j *differ by exactly one nucleotide, then

qij={πj          if i≠j by a synonymous transversionκπj       if i≠j by a synonymous transitionγijωπj  if i≠j by a nonsynonymous transversion  γijωκπj     if i≠j by a nonsynonymous transition            where{γij = 1, if i and j have the same physiochemical propertyγij = γ, if i and j have different physiochemical properties      (2)
 MathType@MTEF@5@5@+=feaafiart1ev1aaatCvAUfKttLearuWrP9MDH5MBPbIqV92AaeXatLxBI9gBaebbnrfifHhDYfgasaacH8akY=wiFfYdH8Gipec8Eeeu0xXdbba9frFj0=OqFfea0dXdd9vqai=hGuQ8kuc9pgc9s8qqaq=dirpe0xb9q8qiLsFr0=vr0=vr0dc8meaabaqaciaacaGaaeqabaqabeGadaaakeaajugGbuaabeqabiaaaqaabeGcbaqcLbyacqWGXbqCkmaaBaaaleaajugGbiabdMgaPjabdQgaQbWcbeaajugGbiabg2da9OWaaiqaaeaajugGbuaabaqaeeaaaaGcbaacciqcLbyacqWFapaCkmaaBaaaleaajugGbiabdQgaQbWcbeaajugGbiabbccaGiabbccaGiabbccaGiabbccaGiabbccaGiabbccaGiabbccaGiabbccaGiabbccaGiabbccaGiabbMgaPjabbAgaMjabbccaGiabdMgaPjabgcMi5kabdQgaQjabbccaGiabbkgaIjabbMha5jabbccaGiabbggaHjabbccaGiabbohaZjabbMha5jabb6gaUjabb+gaVjabb6gaUjabbMha5jabb2gaTjabb+gaVjabbwha1jabbohaZjabbccaGiabbsha0jabbkhaYjabbggaHjabb6gaUjabbohaZjabbAha2jabbwgaLjabbkhaYjabbohaZjabbMgaPjabb+gaVjabb6gaUbGcbaqcLbyacqWF6oWAcqWFapaCkmaaBaaaleaajugGbiabdQgaQbWcbeaajugGbiabbccaGiabbccaGiabbccaGiabbccaGiabbccaGiabbccaGiabbccaGiabbMgaPjabbAgaMjabbccaGiabdMgaPjabgcMi5kabdQgaQjabbccaGiabbkgaIjabbMha5jabbccaGiabbggaHjabbccaGiabbohaZjabbMha5jabb6gaUjabb+gaVjabb6gaUjabbMha5jabb2gaTjabb+gaVjabbwha1jabbohaZjabbccaGiabbsha0jabbkhaYjabbggaHjabb6gaUjabbohaZjabbMgaPjabbsha0jabbMgaPjabb+gaVjabb6gaUbGcbaqcLbyacqWFZoWzkmaaBaaajuaGbaqcLbyacqWGPbqAcqWGQbGAaKqbagqaaKqzagGae8xYdCNae8hWdaNcdaWgaaWcbaqcLbyacqWGQbGAaSqabaqcLbyacqqGGaaicqqGGaaicqqGPbqAcqqGMbGzcqqGGaaicqWGPbqAcqGHGjsUcqWGQbGAcqqGGaaicqqGIbGycqqG5bqEcqqGGaaicqqGHbqycqqGGaaicqqGUbGBcqqGVbWBcqqGUbGBcqqGZbWCcqqG5bqEcqqGUbGBcqqGVbWBcqqGUbGBcqqG5bqEcqqGTbqBcqqGVbWBcqqG1bqDcqqGZbWCcqqGGaaicqqG0baDcqqGYbGCcqqGHbqycqqGUbGBcqqGZbWCcqqG2bGDcqqGLbqzcqqGYbGCcqqGZbWCcqqGPbqAcqqGVbWBcqqGUbGBcqqGGaaicqqGGaaiaOqaaKqzagGae83SdCMcdaWgaaqcfayaaKqzagGaemyAaKMaemOAaOgajuaGbeaajugGbiab=L8a3jab=P7aRjab=b8aWPWaaSbaaSqaaKqzagGaemOAaOgaleqaaKqzagGaeeiiaaIaeeiiaaIaeeiiaaIaeeiiaaIaeeiiaaIaeeyAaKMaeeOzayMaeeiiaaIaemyAaKMaeyiyIKRaemOAaOMaeeiiaaIaeeOyaiMaeeyEaKNaeeiiaaIaeeyyaeMaeeiiaaIaeeOBa4Maee4Ba8MaeeOBa4Maee4CamNaeeyEaKNaeeOBa4Maee4Ba8MaeeOBa4MaeeyEaKNaeeyBa0Maee4Ba8MaeeyDauNaee4CamNaeeiiaaIaeeiDaqNaeeOCaiNaeeyyaeMaeeOBa4Maee4CamNaeeyAaKMaeeiDaqNaeeyAaKMaee4Ba8MaeeOBa4MaeeiiaaIaeeiiaacaaaGccaGL7baajugGbiabbccaGaGcbaqcLbyacqqGGaaicqqGGaaicqqGGaaicqqGGaaicqqGGaaicqqGGaaicqqGGaaicqqGGaaicqqGGaaicqqG3bWDcqqGObaAcqqGLbqzcqqGYbGCcqqGLbqzkmaaceaajugGbqaabeqcaauaaKqzagGae83SdCMcdaWgaaqcbauaaKqzagGaemyAaKMaemOAaOgajeaqbeaakiabbccaGKqzagGaeeypa0JaeeiiaaIaeeymaeJaeeilaWIaeeiiaaIaeeyAaKMaeeOzayMaeeiiaaIaemyAaKMaeeiiaaIaeeyyaeMaeeOBa4MaeeizaqMaeeiiaaIaemOAaOMaeeiiaaIaeeiAaGMaeeyyaeMaeeODayNaeeyzauMaeeiiaaIaeeiDaqNaeeiAaGMaeeyzauMaeeiiaaIaee4CamNaeeyyaeMaeeyBa0MaeeyzauMaeeiiaaIaeeiCaaNaeeiAaGMaeeyEaKNaee4CamNaeeyAaKMaee4Ba8Maee4yamMaeeiAaGMaeeyzauMaeeyBa0MaeeyAaKMaee4yamMaeeyyaeMaeeiBaWMaeeiiaaIaeeiCaaNaeeOCaiNaee4Ba8MaeeiCaaNaeeyzauMaeeOCaiNaeeiDaqNaeeyEaKhajaaqbaqcLbyacqWFZoWzkmaaBaaaleaajugGbiabdMgaPjabdQgaQbWcbeaakiabbccaGKqzagGaeeypa0JaeeiiaaIae83SdCMaeeilaWIaeeiiaaIaeeyAaKMaeeOzayMaeeiiaaIaemyAaKMaeeiiaaIaeeyyaeMaeeOBa4MaeeizaqMaeeiiaaIaemOAaOMaeeiiaaIaeeiAaGMaeeyyaeMaeeODayNaeeyzauMaeeiiaaIaeeizaqMaeeyAaKMaeeOzayMaeeOzayMaeeyzauMaeeOCaiNaeeyzauMaeeOBa4MaeeiDaqNaeeiiaaIaeeiCaaNaeeiAaGMaeeyEaKNaee4CamNaeeyAaKMaee4Ba8Maee4yamMaeeiAaGMaeeyzauMaeeyBa0MaeeyAaKMaee4yamMaeeyyaeMaeeiBaWMaeeiiaaIaeeiCaaNaeeOCaiNaee4Ba8MaeeiCaaNaeeyzauMaeeOCaiNaeeiDaqNaeeyAaKMaeeyzauMaee4CamhaaKaaajaawUhaaKqzagGaeeiiaacaaOqaaKqzagGaaCzcaiaaxMaakmaabmaabaqcLbyacqqGYaGmaOGaayjkaiaawMcaaaaaaaa@EA61@

In both models *q*_*ij *_equals 0 if codon *i *and *j *differ in more than one position, and qii=−∑j≠iqij
 MathType@MTEF@5@5@+=feaafiart1ev1aaatCvAUfKttLearuWrP9MDH5MBPbIqV92AaeXatLxBI9gBaebbnrfifHhDYfgasaacH8akY=wiFfYdH8Gipec8Eeeu0xXdbba9frFj0=OqFfea0dXdd9vqai=hGuQ8kuc9pgc9s8qqaq=dirpe0xb9q8qiLsFr0=vr0=vr0dc8meaabaqaciaacaGaaeqabaqabeGadaaakeaacqWGXbqCdaWgaaWcbaGaemyAaKMaemyAaKgabeaakiabg2da9iabgkHiTmaaqababaGaemyCae3aaSbaaSqaaiabdMgaPjabdQgaQbqabaaabaGaemOAaOMaeyiyIKRaemyAaKgabeqdcqGHris5aaaa@3D9C@. The parameter *κ *is the transition/transversion rate ratio and codon bias is accounted for by incorporating the stationary distribution of codon *j*. Notice that *ω *no longer can be interpreted directly as the nonsynonymous/synonymous (*d*_*N*_/*d*_*S*_) rate ratio, because this ratio also depends on *γ*.

Although it is a gross simplification of the underlying biology to assume that amino acid mutations can be divided into two categories (property altering and property preserving), models based on this simplification provide considerable computational efficiency while allowing the effect of various physicochemical properties to be explored. The original models by [14], and the models by [16] allow a more complex relationship between rate of substitution and physicochemical properties of the amino acids; they do not, however, simultaneously allow for site-specific variation of these properties in the way the models we propose do.

Models A and B are similar in that the new parameter *γ *appears as a product in the rate of codon substitution when the two codons encode for amino acids of different physicochemical properties. In model A, *γ *measures the increase/decrease in the rate of nonsynonymous substitution between codons of different properties compared to the synonymous substitution rate. In this model, *ω *is the nonsynonymous to synonymous substitution rate ratio of codons encoding amino acids with similar physicochemical properties. It is worth noting that when *ω *= *γ *in model A, it is reduced to the original Goldman and Yang 94 model [14]. In model B, however, *ω *accounts for the background nonsynonymous codon substitution rate and hence *γ *is the nonsynonymous substitutions rate between codons encoding for different physicochemical properties compared to the rate of other nonsynonymous substitutions. Once again, model B reduces to the Goldman and Yang 94 model [14] when *γ *= 1.

The two models can be used to address different biological questions. For instance, we can test the hypothesis that the nonsynonymous to synonymous rate ratio between codons encoding for amino acids with different properties is greater than 1 (positive selection) with model A (i.e. testing if *γ*>1). On the other hand, model B can be used to determine if there is an elevated rate of substitution between codons encoding different physicochemical properties compared to the rate of generic nonsynonymous mutations (i.e. testing if *γ*>*ω*). Since our main interest here is to look for positive selection in the more conventional sense (nonsynonymous to synonymous rate ratio >1), we will analyze simulated and real data sets with model A.

We will construct models that allow site-specific variation by assuming that *ω *and *γ *can each have two rate classes: *ω*_0 _= 1, *ω*_1_>1 and *γ*_0 _= 1, *γ*_1 _>1 and therefore, there are 4 possible site classes to which each codon site can belong.

{(i)  :ω0≤1,γ0≤1(ii) :ω0≤1,γ1>1(iii):ω1>1,γ0≤1(iv):ω1>1,γ1>1     (3)
 MathType@MTEF@5@5@+=feaafiart1ev1aaatCvAUfKttLearuWrP9MDH5MBPbIqV92AaeXatLxBI9gBaebbnrfifHhDYfgasaacH8akY=wiFfYdH8Gipec8Eeeu0xXdbba9frFj0=OqFfea0dXdd9vqai=hGuQ8kuc9pgc9s8qqaq=dirpe0xb9q8qiLsFr0=vr0=vr0dc8meaabaqaciaacaGaaeqabaqabeGadaaakeaadaGabaqaauaabeqaeeaaaaqaceaaFRVaeeikaGIaeeyAaKMaeeykaKIaeeiiaaIaeeiiaaIaeiOoaOdcciGae8xYdC3aaSbaaSqaaiabicdaWaqabaGccqGHKjYOcqaIXaqmcqGGSaalcqWFZoWzdaWgaaWcbaGaeGimaadabeaakiabgsMiJkabigdaXaqaaiabbIcaOiabbMgaPjabbMgaPjabbMcaPiabbccaGiabcQda6iab=L8a3naaBaaaleaacqaIWaamaeqaaOGaeyizImQaeGymaeJaeiilaWIae83SdC2aaSbaaSqaaiabigdaXaqabaGccqGH+aGpcqaIXaqmaeGaeaWp7dWs7dWv7dW=+labbIcaOiabbMgaPjabbMgaPjabbMgaPjabbMcaPiabcQda6iab=L8a3naaBaaaleaacqaIXaqmaeqaaOGaeyOpa4JaeGymaeJaeiilaWIae83SdC2aaSbaaSqaaiabicdaWaqabaGccqGHKjYOcqaIXaqmaeaacqqGOaakcqqGPbqAcqqG2bGDcqqGPaqkcqGG6aGocqWFjpWDdaWgaaWcbaGaeGymaedabeaakiabg6da+iabigdaXiabcYcaSiab=n7aNnaaBaaaleaacqaIXaqmaeqaaOGaeyOpa4JaeGymaedaaaGaay5EaaGaaCzcaiaaxMaadaqadaqaaiabiodaZaGaayjkaiaawMcaaaaa@7E00@

Mixture models can then be constructed by allowing each codon site to be in each of these classes with a certain probability, that can be estimated from the data. Likelihood ratio tests (LRTs) can be constructed by comparing models with only a few site classes to models with more site classes. For example, we can test whether selection favors substitution that alter the specified physicochemical properties with the null mixture model A with only two site classes, namely (i) and (iii) (i.e. only allowing the site classes with *γ *≤ 1) against the alternative mixture model A that allows for all four site classes. We refer to these models as model A2 and A1, respectively (Table 1). Various likelihood ratio tests can also be constructed. For instance, we can obtain evidence for the presence of a significant number of sites in site class (ii) (i.e. the null model has site classes (i), (iii) and (iv) against the full model).

Notice that model A1 allows positive selection both on the particular physicochemical property of interest (when *γ*>1) as well as on all other nonsynonymous substitutions that do not alter the physicochemical property of the encoded amino acids (when *ω*>1). In contrast, model A2 does not allow positive selection with respect to the physicochemical property of interest (since *ω*<1). We can set up a likelihood ratio test by comparing twice the log likelihood difference between Model A1 and Model A2 to a χ32
 MathType@MTEF@5@5@+=feaafiart1ev1aaatCvAUfKttLearuWrP9MDH5MBPbIqV92AaeXatLxBI9gBaebbnrfifHhDYfgasaacH8akY=wiFfYdH8Gipec8Eeeu0xXdbba9frFj0=OqFfea0dXdd9vqai=hGuQ8kuc9pgc9s8qqaq=dirpe0xb9q8qiLsFr0=vr0=vr0dc8meaabaqaciaacaGaaeqabaqabeGadaaakeaaiiGacqWFhpWydaqhaaWcbaacbaGae43mamdabaGae4Nmaidaaaaa@3072@-distribution. Here we ask the question if there are some sites with *γ*>1, i.e. whether there is a significant amount of positive selection acting on the physicochemical property of interest.

We make the standard assumptions of the codon-based likelihood framework (e.g. [13]); (1) we assume the true topology of the tree is known and (2) each codon site in the sequence is independent of the others. Then we calculate the log likelihood of the data given the topology of the tree and the model by summing up the log-likelihood of each site. The likelihood is optimized by the BFGS algorithm in Numerical Recipes in C [17]. After the maximum likelihood estimates (MLEs) are obtained, we use the Empirical Bayes approach [12] to assign sites to site classes. The source code of the C program which implements both of the models (EvoRadical) has been deposited to sourceforge.net [18] and is licensed under the GPL [19].

### Data analysis

**Figure 1:**
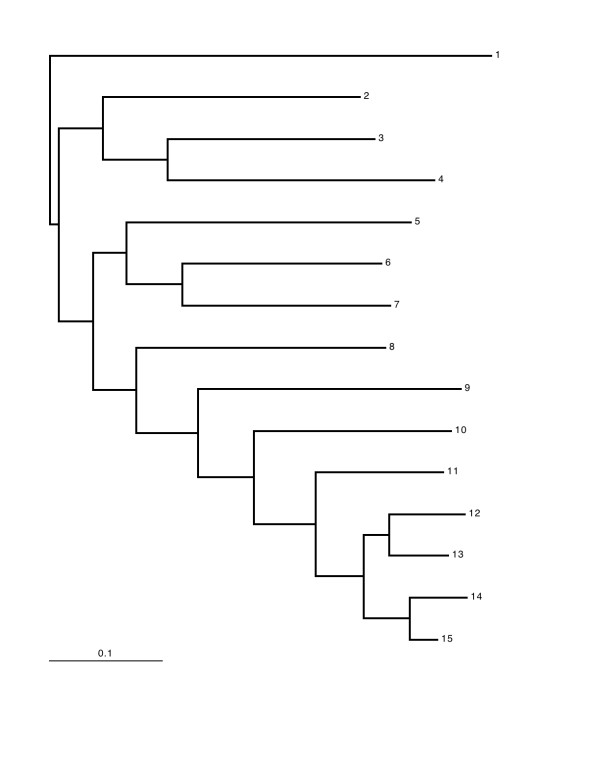
The 15 taxa tree used in the simulation study.

In order to investigate the performance of the model we analyze both simulated and real data sets. The simulated data sets were generated using a modified version of Evolver in the PAML 3.13d package [20]. The parameters used for simulation were *ω *= 0.4 and *γ *= 4 for data set 1, *ω *= 4 and *γ *= 4 for data set 2, *ω *= *γ *= 0.4 for data set 3 and *ω *= *γ *= 4 for data set 4. The transition/transversion ratio *κ *was set to 4 for all data sets. We used the volume partition for all four simulated data sets, by dividing amino acids into 2 groups: large volume (I, L, M, F, Y, Q, W, H, K, R, E), and small volume (V, A, G, P, T, C, S, N, D). A 15 taxa tree was used (Figure 1). We analyzed the data with both volume and hydrophobicity partitions under both Models A1 and A2 using the previously described methods, and obtained maximum likelihood estimates of the branch lengths of the tree, *ω*_0_, *ω*_1_, *γ*_0_, *γ*_1_, *κ*, the proportions of each category, and the posterior probabilities of each site belonging to each site class. The results are summarized in Table 2.

We re-analyzed the MHC class I data set from [21] ([22,23]) with Model A. [21] found numerous positively selected sites and showed that most of them are Antigen Recognition Sites (ARS). The same data set was also used in [11], to examine if the new method identifies the same sites as the original method. We also analyzed the abalone sperm lysin data from [23], with regard to the hydrophobicity, volume, polarity and charge physicochemical properties. We specified each of the four physicochemical properties: volume, hydrophobicity, charge, and polarity partitions with both Model A1 and A2 in these analyses.

#### Simulated data

**Figure 2:**
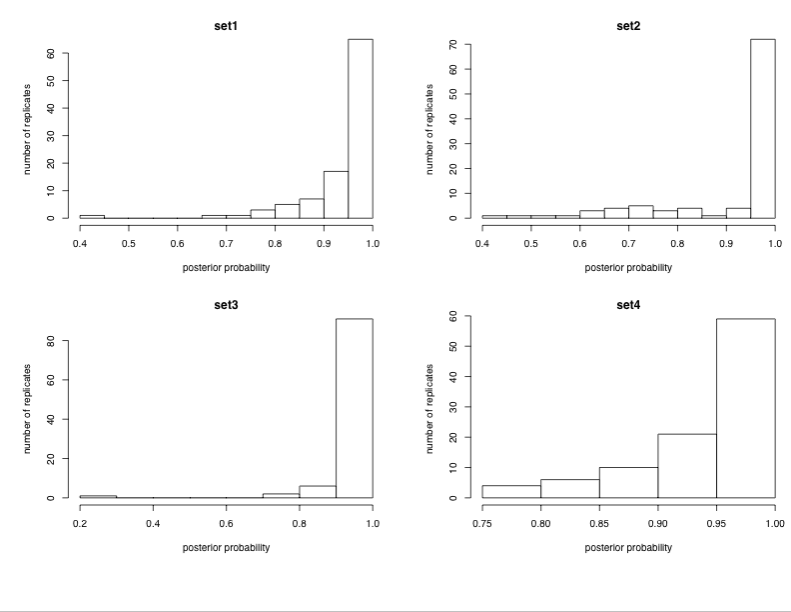
**Distribution of the posterior probabilities for the correct site class classification in each of the simulated data sets**. Each data set has 100 replicated and they were analyzed using Model A1 and the volume partition.

The result of the simulation analysis is summarized in Table 2 and Figure 2. When the correct partition was used, both the LRT and the posterior Bayesian categorization were very accurate. When simulated data set 1 (with *ω*≤1 and *γ*>1) was analyzed using the volume partition (the same partition that the data was simulated from) and there was positive selection acting only on codon substitutions that altered the volume of the encoded amino acids, 99% of the sites were correctly identified as being in the *ω*≤1, *γ*>1 category. Moreover, the LRTs performed between Model A1 versus Model A2 on the 100 replicates were all significant. When the data was simulated with *ω*>1 and *γ*<1 (data set 2), 97% of the sites were classified in the correct site class, and none of the LRTs between Models A1 and A2 were significant, as expected.

Amino acids that differ in volume may also differ in other physicochemical properties. Positive selection on another physicochemical property may then be inferred, even though selection may have truly targeted volume. To illustrate this concept and better understand its effect, we repeated the data analysis with other physicochemical properties. We partitioned the amino acids into those with high (A, C, I, V, L, M, F, Y, W, H, T, K, R) and low (R, G, S, D, E, N, Q) hydrophobicity. In each replicate, data was simulated assuming selection only targeted volume, but was analyzed under the hydrophobicity partition. In this case the resulting LRTs were all significant for both data set 1 and data set 2 (*ω*>1 and *γ*>1). The results were significant for data set 1 when the hydrophobicity physicochemical property was used due to some volume altering codon changes that are hydrophobicity altering as well (52 out of the 99 distinct amino acid pairs that differ in volume). Likewise, since there are some volume conserving nonsynonymous codon changes that are hydrophobicity altering (44 out of the 91 distinct amino acid pairs that differ in hydrophobicity), the LRTs were significant for data set 2 as well. Thus, the extent of overlap between two sets of distinct amino acid pairs, where each set contains pairs that differ with respect to one of the two given physicochemical properties, ultimately determines our ability to distinguish between the two properties, in terms of being exclusively targeted by positive selection. Nonetheless, our method can reliably identify the true physicochemical property in addition to other properties that have considerable overlap with the true property.

Data sets 3 and 4 were simulated with *ω *= *γ*, in which case our model reduces to the original Goldman and Yang 94 model [14]. Our method classified = 99% of the sites correctly with respect to both volume and hydrophobicity partitions for both data set 3 (*ω*≤1 and *γ*≤1) and data set 4 (*ω*>1 and *γ*>1).

Figure 2 shows the distribution of the posterior probabilities for the correct site class classification in each of the data sets. The vast majority of replicates have posterior probabilities close to one indicating that assignments of the sites into their site classes can be done with high confidence.

#### The MHC class 1 dataset

Table 3 shows the log-likelihoods and the parameter estimates of the analysis of the MHC class 1 dataset. The LRTs of Model A2 against Model A1 were significant in all the partitions examined; indicating that a significant amount of positive selection is acting on the codon substitutions that change the physicochemical property under study. We also found that, for each partition, there are a fair proportion of sites that are in the (*ω*<1, *γ*>1) category, suggesting positive selection acting on the particular physicochemical property.

However, when we looked at the sites that were identified in the previous study [11] with high posterior probabilities of being positively selected with respect to a particular physicochemical property; we found that some of these sites were positively selected regardless of the physicochemical property of the amino acids they code for (Table 4). For instance, almost all the posterior probability mass of the 3 sites that were identified for positive selection with respect to the volume partition (63, 67, 93) were more likely to be in the categories with *ω*>1; and the posterior probabilities of being in the (*ω*≤1, *γ*>1) category were almost 0. The same was observed for the polarity partition. Nevertheless, the posterior probabilities of being in the (*ω*≤1, *γ*>1) category were relatively high at sites 45,114 and 156 (sites that were previously identified to be positively selected with respect to charge), although they were still more likely to be in the (*ω*>1, *γ*>1) category. The main reason for this discrepancy between our study and that of [11] is due to the manner in which physicochemical selection is defined and measured. [11] defined the parameter gamma to be the rate of nonsynonymous substitutions that alter the property of the encoded amino acids and scale it relative to the rate of all other codon substitutions pooled together, i.e. both synonymous substitutions and property-conserving nonsynonymous substitutions. However, we extended their model to allow for a separation of the pooled scaling rate into rates of nonsynonymous property-conserving substitutions and synonymous substitutions. This naturally implies that our parameter gamma is not equivalent to their gamma even if the underlying physicochemical property is the same. However, our parameterization is biologically more realistic since it allows a more sensible scaling with respect to the synonymous substitution rate.

#### The abalone sperm lysin dataset

The results of the analysis of the abalone sperm lysin data, when amino acids are categorized according to hydrophobicity, volume, polarity and charge are shown in Table 5.

##### 1) The charge partition

Our method identified sites belonging to all 4 classes with high posterior probabilities. We compared with the results obtained by [23] (M3 in Table 4) using the PAML software [20]. We found that all sites in the (*ω*≥1, *γ*≥1) site class were also classified as positively selected sites (posterior probabilities > 0.95) by [23] using model M3 of the standard framework. On the other hand, none of the sites in the *ω*<1 site classes (*ω*<1, *γ*<1 and *ω*<1, *γ*≥1) were classified as positively selected in their study. Thus, our results identified additional categories of putatively positively selected sites in which selection seems to have favored substitutions that alter specific physiochemical properties of the amino acids.

**Figure 3:**
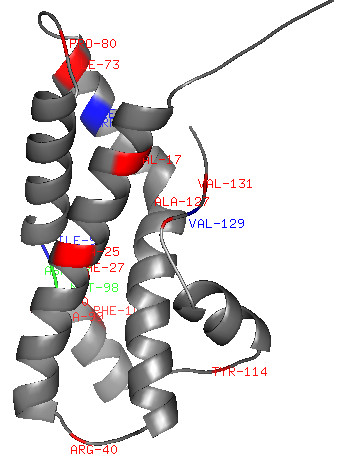
**Lysin crystal structure from the red abalone Haliotis rufescens ([24], PDB ID 1ILS)**. Sites in color are in the (*ω*≤1, *γ*≥1) category. Sites that are blue (68,69,96,129) are from the volume and charge partitions. Sites that are red (17,5,27,30,40,73,80,99,101,114,127,131) are from the volume partition only. Finally, sites that are green (97–98) are from the charge partition only.

Figure 3 illustrates that positively selected sites tend to cluster together in the 3-dimensional protein structure. Although some sites in the (*ω*≥1, *γ*<1) site class were identified previously as being positively selected, there are 12 sites (Sites 30, 47, 63, 71, 75, 79, 80, 81, 99, 116, 121 and 124) that were not (See Figure 3). These sites were probably under positive selection as well but under the constraint that they had to maintain the same charge. Our method may have more power than the original model to identify positively selected sites when positive selection is only targeting a particular physiochemical property while the site remains conserved with respect to other properties.

##### 2) The volume partition

**Figure 4:**
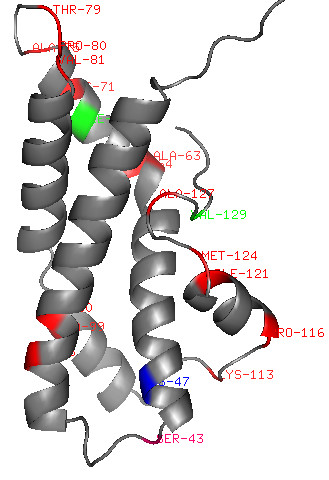
**Lysin crystal structure from the red abalone Haliotis rufescens ([24], PDB ID 1ILS)**. Sites in color are in the (*ω*≥1, *γ*≤1) category. The site that is blue (47) from both the charge and hydrophobicity partitions. Sites that are green (69, 129) are from the hydrophobicity partition only. Sites that are red (30,33,63,64,71,75,79,80,81,99,113,116,121,124,127) are from the charge partition only. Finally, the site that is hot pink (43) is from the polarity partition only.

There are a number of sites that are targeted for positive selection with respect to the volume property (*ω*≤1, *γ*>1), however none of the positively selected sites are conserved under this partition. This may indicates that volume is an important property targeted by positive selection. It is worth mentioning that most of the sites in the (*ω*≤1, *γ*>1) site class are adjacent in the 3D structure to a positively selected site previously identified by [23]. For instance, sites 40, 73, 114, 131 are adjacent to sites 41, 74, 113, 132 (Figure 4), and site 127 is adjacent to 126. It is possible that, as [11] pointed out earlier on the basis of the vast site-directed mutagenetic literature, substitutions involving volume change of the residues may slightly change the structure of the peptide which in turns improves the ability to incorporate new mutations.

## Discussion

A major limitation of the current approach is that the correct partition cannot be directly inferred from the data. The method cannot statistically identify which amino acid property is being targeted by positive selection without input from the user, however, the program can be used to explore pre-defined hypotheses regarding positive (or negative) selection. Our simulation study showed that when the correct partition has been specified, the method can accurately detect positive selection acting on the associated amino acid property.

We note that the similarities between amino acids are probably better described by continuous functions. Much work has been done on continuous models (e.g. [14,16]) in the context of codon-based likelihood models. However, in the framework of mixture models that allow variation across sites, these approaches are not computationally tractable and require a discrete approximation over many categories in multiple dimensions. Our new method provides more information than previous methods and yet maintains computational efficiency.

We also want to note that the regularity conditions for the *χ*^2 ^approximation are not satisfied with the LRT between A2 and A1. This is because when either of the parameters *p*_3 _or *p*_4 _hit the boundary of the parameter space (i.e. when *p*_3 _= 0 or *p*_4 _= 0), either *ω*_1 _or both *ω*_1 _and *γ*_1 _is non-identifiable.

It is worth mentioning that a significant LRT does not necessarily imply that there is positive selection acting solely on the physicochemical property defining the partition examined. Since the amino acids that differ with respect to physicochemical property may also differ with respect to other properties, it is not possible with the current method to exclude that selection has been targeting other properties over the one specified by the user.

Positive selection inferred by the new method informs the user that there are more substitutions between amino acid in the chosen partition than expected under neutral evolution. A clear advantage of our method over methods that do not take physicochemical properties into account is that it can detect positive selection when selection is only acting on some particular subset of nonsynonymous substitutions, while conserving others. Thus, positive and negative selection acting on the same protein residue can be inferred simultaneously for any specified physicochemical property in a site-specific manner.

This method can also be used to examine the pattern of negative selection in proteins that may not be under positive selection. In many cases, it would be interesting to explore which residues are conserved predominantly with respect to a particular physicochemical property. Site specific models of physicochemical selection provide a more statistically rigorous framework for finding sites that are under selection than method that are based on calculating amino acid content in particular sites without taking the underlying phylogenetic tree into account.

## Conclusion

We see this new method as a step forward in incorporating information regarding physicochemical properties into studies of positive selection, as well as a step towards methods that allow identification of selection acting on particular amino acid properties, without any prior specification by the user of which properties to examine. Models that allow site-specific selection on different amino acid properties will be an important tool in studies of molecular evolution and should help bridge the gap between structural biology and molecular evolution.

## Availability and requirements

Project name: EvoRadical

Project website: 

Operating System(s): Tested on Linux and Mac OS X.

Programming language: ANSI C

License: GPL

Non-academic licensing: None

## Authors' contributions

WSWW wrote the program EvoRadical, performed the analysis, drafted and finalized the manuscript. RN supervised the project and RN and RS contributed to the modeling and writing of the manuscript. All authors have read and approved the manuscript.

## Figures and Tables

**Table 1:** Mixture models of *ω *and *λ*

Model	Site classes*	parameters	constraints	P**
A1	(i), (ii), (iii), (iv)	*ω*_0_, *ω*_1_, *γ*_0_, *γ*_1_, *p*_0 _*p*_1_, *p*_2_	*ω*_0_≤1, *ω*_1_>1, *γ*_0_≤1, *γ*_1_>1, *p*_3 _= 1-*p*_0_-*p*_1_-*p*_2_	7
A2	(i), (iii)	*ω*_0_, *ω*_1_, *γ*, *p*_0_	*ω*_0_≤1, *ω*_1_>1, *γ*≤1	4

* With regard to the site classes listed in Equation (2)

** P = number of parameters in the *ω *and *γ *distributions

**Table 2:** Summary of the results from simulated data under Model A1. The Likelihood Ratio Tests were performed between Model A1 versus Model A2 using the volume partition at the 5% significance level. The table also shows the percentage of sites predicted to be in each site class.

Data Set	Partition	% of significant LRT tests*	% of sites in each category			
			
			*ω*≤1,*γ*≤1	*ω*≤1,*γ*>1	*ω*>1,*γ*≤1	*ω*>1,*γ*>1
Set 1 (*ω *= 0.4,*γ *= 4.0)	Volume	100	0.00	**0.99**	0.00	0.01
	Hydrophobicity	100	0.27	0.41	0.08	0.24
Set 2 (*ω *= 4,*γ *= 0.4)	Volume	0	0.00	0.00	**0.97**	0.03
	Hydrophobicity	100	0.08	0.11	0.17	0.64
Set 3 (*ω *= *γ *= 0.4)	Volume	4	**0.99**	0.00	0.00	0.01
	Hydrophobicity	1	**0.99**	0.00	0.01	0.00
Set 4 (*ω *= *γ *= 4)	Volume	100	0.00	0.00	0.00	**1.00**
	Hydrophobicity	100	0.00	0.00	0.00	**1.00**

* Bold numbers indicate the correct category

**Table 3:** Log-likelihood Values and Parameter Estimates of the different physicochemical properties for the MHC data.

Data set/Model	loglikelihood	*κ*	*ω*	*γ*	proportions
MHC Class I					
***Volume***					
Model A1	-2463.35	2.86	0.22, 8.74	0.00, 3.47	Prob (*ω*_0_, *γ*_0_) = 0.73
					Prob (*ω*_0_, *γ*_1_) = 0.17
					Prob (*ω*_1_, *γ*_0_) = 0.07
					Prob (*ω*_1_, *γ*_1_) = 0.03
Model A2	-2474.61	2.84	0.23, 9.30	0.53	Prob (*ω*_0_, *γ*_0_) = 0.90
					Prob (*ω*_1_, *γ*_0_) = 0.10

***Hydrophobicity***					
Model A1	-2464.89	2.81	0.14, 9.94	0.12, 5.48	Prob (*ω*_0_, *γ*_0_) = 0.80
					Prob (*ω*_0_, *γ*_1_) = 0.09
					Prob (*ω*_1_, *γ*_0_) = 0.07
					Prob (*ω*_1_, *γ*_1_) = 0.04
Model A2	-2505.65	2.59	0.09, 9.12	0.62	Prob (*ω*_0_, *γ*_0_) = 0.87
					Prob (*ω*_1_, *γ*_0_) = 0.13

***Charge***					
Model A1	-2470.99	2.75	0.00, 5.46	0.19, 5.48	Prob (*ω*_0_, *γ*_0_) = 0.76
					Prob (*ω*_0_, *γ*_1_) = 0.05
					Prob (*ω*_1_, *γ*_0_)) = 0.11
					Prob (*ω*_1_, *γ*_1_) = 0.08
Model A2	-2515.343693	2.59	0.37,18.77	0.65	Prob (*ω*_0_, *γ*_0_) = 0.94
					Prob (*ω*_1_, *γ*_0_) = 0.06

***Polarity***					
Model A1	-2470.149113	2.93	0.17, 6.99	0.02, 3.85	Prob (*ω*_0_, *γ*_0_) = 0.75
					Prob (*ω*_0_, *γ*_1_) = 0.12
					Prob (*ω*_1_, *γ*_0_) = 0.04
					Prob (*ω*_1_, *γ*_1_) = 0.08
Model A2	-2491.533373	2.89	0.18,7.75	0.61	Prob (*ω*_0_, *γ*_0_) = 0.88
					Prob (*ω*_1_, *γ*_0_) = 0.12

**Table 4:** Posterior probabilities of being in each site class for the previously identified positively selected sites in the MHC data in [11].

	Previously identified sites	Posterior probabilities in the 4 categories with the same partition			
		*ω*_0_<1, *γ*_0_<1	*ω*_0_, *γ*_1_	(*ω*_1_, *γ*_0_)	(*ω*_1_, *γ*_1_)
Volume-altering	63	0.00	0.00	0.72	0.28
	67	0.00	0.03	0.60	0.37
	97	0.00	0.00	0.01	0.99

Polarity	116	0.00	0.00	0.01	0.99

Charge	45	0.00	0.28	0.00	0.72
	114	0.00	0.39	0.00	0.61
	156	0.00	0.36	0.00	0.64

**Table 5:** Abalone sperm lysin data [23] analyzed with Model A1 and 4 (hydrophobicity, volume, polarity and charge) partitions: sites that have high posterior probabilities in each site class.

Property	Sites identified			
	
	*ω*≤1, *γ*≤1	*ω*≤1, *γ*≥1	*ω*≥1, *γ*≤1	*ω*≥1, *γ*≥1
hydrophobicity	16, **20**, **23**,26, 31,**34**,39,**48**, **52**,**54**,**55**,57, **58**,**59**,60,**62**, **65**,66,77,**78**,84, **85**,89,90,**91**,**92**, 93,**102**,111,**112**, **118**	none	**47**,69,129	**10,12,32,33,36**, **37**,40,**44**,45,64, **67**,**68**,70,**72**,**74**, 82,**83**,86,87,106, **107,113**,116, **120**,123,127
volume	13,**18**,19,**23,29**, 35,**39**,50,51,53, 54,**55,56**,57,65, 76,**77,78**,85,89, **90**,**92,93,94,95**, 102,**111,112**,117	17,25,27,30,40, **68,69,73,80,96**, 99,101,**114**, **127**,129,131	none	11,**32**,33,36,**41**, 64,**70**,74,**83**,**86**, 87,**120**
polarity	16,18,20,23,26, 28,31,34,35,38, 39,46,48,50,52, 55,56,57,58,59, 60,62,65,66,76, 77,78,84,85,89, 90,91,92,93,94, 95,102,104,112, 118,128,130	none	43	**10**,11,**12**,**14**,15, **32**,**33**,**36**,37,**40**, **44,64,67,70**,**74**, 79,**83,86**,87,**106**, 116,**120**,123,**127**
charge	**16,20,23**,28,29, **46,48,52,54,59**, **65**,84,**85**,**91,92**, **102**,104,109,111, **112**,117,**118**,128	**68**,69,96,97,98, **129**	30,33,**47**,**63**,64, **71**,**75**,79,80,81, **99**,113,**116,121**, **124**,127	**10**,14,**44**,67, **70**,74,**115**,120

Site listed have posterior probabilities >0.95 being in the indicated site class. Those that are in bold have posterior probabilities >0.99.
